# Case of Procidentia Uteri; with Remarks

**Published:** 1830-01-01

**Authors:** 


VIII.

CASE OF PROCIDENTIA UTERI. WITH REMARKS. By J. J. KNOX.*

E. Stivens, æt. 20, unmarried, of delicate habit, applied to Mr. Knox with a tumour in the vagina, protruding between the labia, about four inches in length, of a deep red colour, and excoriated in a high degree. She complained of general debility; great pain in the back and loins and constant bearing down sensation; much inconvenience in walking; and pain in voiding her urine which she could not retain so long as she used to do. She stated that the tumour first made its appearance three months previous to her application, that then it was small, and had gradually increased to its existing size, and that she had been much troubled with leucorrhœa. A more minute examination was now instituted. The finger could not be introduced into the vagina, nor could the os tincæ be felt, but on carefully inspecting the apex of the tumour a small foramen was discovered, which easily admitted the blunt end of a probe, and from which a red liquor, evidently the catamenial discharge, then present, was oozing. The lips of the os tincæ were completely obliterated in consequence of the swelling of the parts, and presented a circumference the size of a dollar, in the centre of which the orifice into the uterus was placed. The case was obviously one of procidentia uteri, and the complete eversion of the vagina accounted for the impossibility of introducing the finger.

Much to Mr. Knox's surprise, gentle pressure on the tumour in the line of the axis of the pelvis, the patient being placed upon her back with the hips elevated, readily effected the return of the prolapsus. Next morning a pessary was introduced, and answered the intention well in conjunction with injections of alum and oak-bark. The discharge ceased, the uterus descended no more, and the patient experienced no inconvenience from the instrument.

"Since I met with this case, I have had an opportunity of examining more minutely the state of the parts in this disease, in the body of an old woman, which I was requested to open. In her, a tumour nearly five inches in length, and nine or ten in circumference, protruded between the thighs, presenting all the external characters noticed in the above. Being anxious to have a view of the parts within the pelvis, I opened the abdomen for that purpose. The protruded vagina formed a large cul-de-sac, in which were contained the uterus and its appendages, the bladder and part of the small intestines. The parts were much altered and thickened by inflammation, and bound together by innumerable bands of new membrane, which could with difficulty be separated by the fingers. The bladder was much smaller than usual.

"The everted vagina was not unlike to skin, and when cut into, was found to be nearly half an inch in thickness, and remarkably hard.

"Procidentia uteri is, comparatively speaking, a rare disease in young and unmarried females. It would appear, that whatever tends to relax or dilate the passages, gives a tendency to this most troublesome complaint; and hence it most frequently happens to those who have had large families, and who have been much troubled with leucorrhœal discharges. It is very trouble-

* Glasg. Journ. No. VII.

some, and not unfrequently incurable. The urine cannot be retained so long as usual, which is easily accounted for; as in the dissection related above, the fundus vesicæ was dragged along with the uterus, and retained in its new situation by membranous bands, which prevented its distention; its passage, also, along the tumour, produces excoriation and great uneasiness. In the treatment of this formidable disease, it is of the utmost importance, as soon as its nature is known, to replace, and retain the parts in their natural situation; but it not unfrequently happens, that if they have been long displaced and unattended to, it is impossible to do so, and dangerous to persist in our attempts at reduction, if great difficulty is experienced. In the dissection related above, the parts were not only altered in texture, and consequently would, if reduced, have operated as a foreign body, but were so bound down by adhesions, as would have prevented their reduction, or, if ruptured, have occasioned such a degree of inflammation as in the end would have been fatal. In old and irreducible cases, therefore, the best and only thing that can be done, is to support the displaced parts, in order to guard against their farther descent, and to protect them from injury.

"Another interesting fact derived from the above case is the confirmation of the already received opinion, that the catamenial discharge is elaborated by the uterus, and not, as was formerly imagined, by the vagina, as in this case the secretion was seen oozing from the mouth of the uterus, so that, added to those related by Morgagni, and Dr. William Hunter, it sufficiently proves that the catamenia are secreted by the uterus alone."

